# EEG synchronization patterns during a Go/No-Go task in individuals with aphasia in subacute and chronic phases of stroke

**DOI:** 10.1038/s41598-024-75259-7

**Published:** 2024-10-10

**Authors:** Jacek Rogala, Mateusz Choinski, Aneta Szymaszek

**Affiliations:** 1 Centre for Systemic Risk Analysis, University of Warsaw, 26/28 Krakowskie Przedmiescie Street, 00-927 Warsaw, Poland; 2 Laboratory of Neurophysiology of Mind, Nencki Institute of Experimental Biology, Polish Academy of Sciences, 3 Pasteur Street, 02-093 Warsaw, Poland; 3 Faculty of Psychology, University of Warsaw, 5/7 Stawki Street, 00-183 Warsaw, Poland

**Keywords:** Aphasia, EEG coherence, Inhibition, Post-stroke phases, Synchronized oscillations, Human behaviour, Cognitive neuroscience, Neuronal physiology

## Abstract

Stroke and subsequent neuroregenerative processes cause changes in neural organization of attentional functions. In this study, we attempted to identify differences in neural synchronization patterns during a visual Go/No-Go task in people with post-stroke aphasia in both subacute and chronic stroke phases. To identify neuronal underpinnings of the behavioral differences we investigated pairwise connectivity patterns using corrected imaginary phase locking value and graph-theoretic measures (efficiency, modularity and clustering coefficient) at global and local level in subacute (*n* = 13) and chronic stroke phases (*n* = 14) during a Go/No-Go task. We observed significantly lower phase synchronization in the Subacute Group in the alpha band in the connections spanning frontal and central areas of both hemispheres alongside lower local efficiency and clustering coefficient in the left frontal region. Additionally, we observed higher modularity in the beta band in the unaffected right parietal region in the Subacute Group which may denote inhibition of motor and attention functions. Those mechanisms could serve to align cognitive abilities between the damaged and healthy hemispheres, harmonizing the activity of the neuronal networks of both hemispheres disrupted by the effects of the stroke. Our findings have potential implications for rehabilitation therapies, which should take into account the pattern of connectivity changes during different phases of reovery.

## Introduction

Stroke is the third leading cause of death and disability in the world^1^. About one third of stroke survivors suffer from aphasia (i.e., linguistic deficits following a lesion to the language-dominant hemisphere^2^). Depending on the localization of the lesion, people with aphasia (PWA) struggle with deficient speech production and/or comprehension, which is pervasive over a one-year period in 61% of cases^3^.

While the most salient deficits in PWA concern the linguistic domain, impairments of other cognitive functions are also observed. PWA present with deficient working and short-term memory^4,5^, executive functions^6^, temporal information processing^7,8^, as well as attention^9,10^. These non-linguistic deficits further disturb communication abilities in PWA and hinder linguistic therapy and the recovery process. Novel therapeutic methods addressing non-linguistic functions have therefore been developed for PWA^11–14^.

Attention is one of the functions most commonly reported to be deficient in PWA. The prevalence of attention deficits following stroke is reported to vary from 46 to 92%^15^. Attention, as a limited-capacity resource, is needed for correct execution of most complex mental processes^10^. According to von Steinbüchel and Pöppel’s^16^ classification of cognitive functions, attention may be considered a logistic function, providing a neuronal template for content-related functions, such as memory, perception, and language. Thus, efficient attention is a prerequisite for effective linguistic performance. Some authors even suggest that linguistic deficits in aphasia are secondary to attention deficits^17^. This hypothesis requires further study; however, it has been shown that attention deficits often accompany aphasia^9^. The attention deficits in aphasia seem domain-general, as they can also be observed in tasks that use non-linguistic stimuli^15,18^.

It is worth noting that attention deficits in PWA pertain to different types of attention^10^, potentially making it difficult to compare results across studies. Spaccavento and colleagues^15^ reported a distinction between intensive processes (alertness and vigilance) and selective processes (focused and divided attention). In PWA, deficits in selective processes seem more prevalent than in intensive processes^18^. Among people after left-hemispheric stroke, the presence of aphasia is associated with deficits in selective attention assessed with a visual Go/No-Go task. Overall, 57% of PWA (versus 10% of non-aphasics) displayed deficient reaction time in the Go/No-Go task. The authors consider the possible engagement of the phonological loop, thus verbalization, which is deficient in PWA, during the retention of abstract shapes in memory^15^.

The study of Schumacher et al.^18^ reported that, depending on the type of attention assessed, from 3 to 53% of PWA in the chronic phase (at least a year after stroke) displayed deficits compared to healthy people. Spaccavento et al.^15^ reported a smaller proportion (ca. 15%) of PWA displaying specifically deficits in selective attention and inhibition (i.e., decreased reaction time on a Go/No-Go task). These discrepancies in proportions may be caused by the selection of patients for the studies. In addition to PWA in the chronic phase, Spaccavento et al. also included patients in the subacute phase, in whom cognitive deficits, including deficient attention, may be more severe, as the brain is still in a dynamic process of recovery from the stroke.

Moreover, studies have revealed that attentional dysfunctions may be associated with distinct lesion localizations depending on attention type, with inhibition being related to left frontal areas. Recovery from aphasia and the accompanying non-language cognitive deficits with its neural mechanisms is strongly related to stroke phase. The crucial period for aphasia rehabilitation seems to be the first six months after the stroke onset (subacute phase^19,20^), during which spontaneous recovery of brain functions occurs; this may facilitate therapeutic intervention effects as the recovery process is more dynamic^21^. While recovery after that period (chronic phase) still occurs, its scope is typically smaller^2^. The neural reorganization supporting recovery seems distinct at these two phases. In the subacute stroke phase, PWA display reduced activation in the damaged left hemisphere and compensate with increased activation of healthy right-hemispheric homologues^22^. The recruitment of the right hemisphere is associated with better linguistic recovery in this phase. In the chronic phase, the reparatory processes are substantially slower; however, further neuroplasticity changes occur due to environmental factors, such as language therapy^21^. Heiss and Thiel^23^ indicate that the most successful language recovery follows the recruitment of residual functional left-hemispheric areas. Although the most striking effects of stroke affecting the left hemisphere are associated with aphasia, the damage also affects other higher order functions such as attention. Unfortunately, little is known about reorganization of brain activity associated with left hemisphere lesions and the accompanying recovery processes.

The current study aimed to explore the differences in neural reorganization associated with stroke phase (subacute vs. chronic patients). The most direct measure of such reorganizations is changes in functional connectivity. Functional connectivity refers to the synchronized activity amongst disparate neural assemblies, which facilitates the accomplishment of complex cognitive tasks or perceptual processes^24^. Using EEG methodology, we can quantify this synchronization through various measures, including frequency and phase spectra, topographic maps, standing and traveling waves, as well as covariance and coherence within bi- or multi-variate structures^25^. Anatomical and functional networks of the brain are shown to be organized between a regular grid and random connections^26^, creating dense or clustered local connectivity with relatively few long-range connections mediating a short path between any pair of neurons or regions in the network^27^. In contrast, the community structure of brain networks is modular, with each module consisting of many densely connected regional nodes, and each node often shares functional specializations and/or anatomical locations with other nodes in the same module. Thus, clustering and modularity are related topological properties that favor specialized or segregated information processing in brain networks^28^. However, cognitive abilities depend on the consolidation of long-distance connections that are necessary to break modularity and that support the emergence of highly efficient networks. Investigating synchronization and brain networks organization patterns in lesioned brains informs us about reorganization following stroke while performing attentional tasks and neural changes depending on stroke phase. Our investigation focused on functional brain connections in PWA, during both subacute and chronic phases, while they performed an electrophysiological Go/No-Go task. We primarily compared the strength of pairwise phase synchronizations and following patterns of differences in brain organization at subacute and chronic phase of recovery. To our knowledge, this constitutes the first study investigating functional brain synchronization in PWA patients across different stroke phases while engaging in an attentional task; we therefore decided to adopt an exploratory approach. The majority of prior electrophysiological studies assessing brain connectivity during the execution of Go/No-Go tasks have typically involved healthy subjects, making our study a unique contribution to the field.

## Methods

### Participants

A total of 27 post-stroke patients (18 male) suffering from aphasia after the first left-hemispheric stroke (lesion age: *M* ± *SD* = 47 ± 49 weeks) participated in the study. Participants’ ages ranged from 40 to 78 years (*M* ± *SD* = 60 ± 12 years). They were right-handed native speakers of Polish. Participants displayed normal hearing levels verified by pure-tone screening audiometry (Audiometer MA33, MAICO). The following exclusion criteria verified during an interview with the caregivers of the patients were applied: recurrent stroke, global aphasia, severe comprehension impairment, post-stroke visual deficits, prior neurological or psychiatric diseases, substance abuse, history of head injuries, and signs of dementia. Participants were divided into two groups depending on the time from stroke onset—a Subacute Group (lesion age shorter than 6 months, *n* = 13) and a Chronic Group (lesion age equal or longer than 6 months, *n* = 14).

The detailed characteristics of the patients are presented in Table 1 and the comparison between groups is displayed in Table 2. Mean age did not differ between PWA from the Subacute and Chronic Groups; however, participants in the chronic phase displayed significantly larger lesion volume (independent samples t-test: *t*(20) = −2.42; *p* = 0.025). There was also a significantly greater percentage of female participants in the Chronic Group.

**Table 1:** Characteristics of the patient sample (abbreviations: M—male; F—female; n.a.—not available, I—ischemic stroke; H—hemorrhagic stroke).

ID	Age (years)	Sex	Lesion age (weeks)	Lesion volume	Type of stroke	Group
1	60	M	8	n.a.	I	Subacute
2	69	M	21	56,011	I	
3	74	M	6	n.a.	I	
4	78	M	24	3740	I	
5	78	M	5	n.a.	I	
6	51	M	12	42,429	I	
7	74	M	6	37,303	I	
8	59	M	15	22,798	I	
9	71	M	20	140,928	I	
10	64	M	10	84,121	I	
11	46	M	18	17,216	I	
12	48	M	9	45,655	I	
13	48	F	20	n.a.	I	
14	58	M	169	53,182	I	Chronic
15	40	M	73	95,664	I	
16	60	M	81	73,481	I	
17	67	M	70	195,985	I	
18	43	M	49	121,210	I	
19	62	M	33	76,228	H	
20	58	F	72	93,033	I	
21	54	F	194	103,136	I	
22	62	F	31	56,184	I	
23	51	F	37	44,781	H	
24	75	F	47	n.a.	I	
25	44	F	25	98,472	I	
26	49	F	96	71,275	I	
27	72	F	114	111,584	I	

**Table 2:** Comparison of demographic and stroke-related variables between PWA from Subacute and chronic Groups.

	Subacute Group (*n* = 13) M (SD)	Chronic Group (*n* = 14) M (SD)	Comparison
Age (years)	63.08 (11.91)	56.79 (10.64)	*t*(25) = 1.45 *p* = 0.16
Lesion age (weeks)	13.38 (6.6)	77.93 (51.15)	*U* = 0.0 *p* < 0.001
Lesion volume (mm^3^)	50022.33 (41322.53)	91862.69 (39019.52)	*t*(20) = −2.42 *p* = 0.025
Gender (male/female)	12/1	6/8	χ^2^(1) = 7.42 *p* = 0.006

**Fig. 1:**
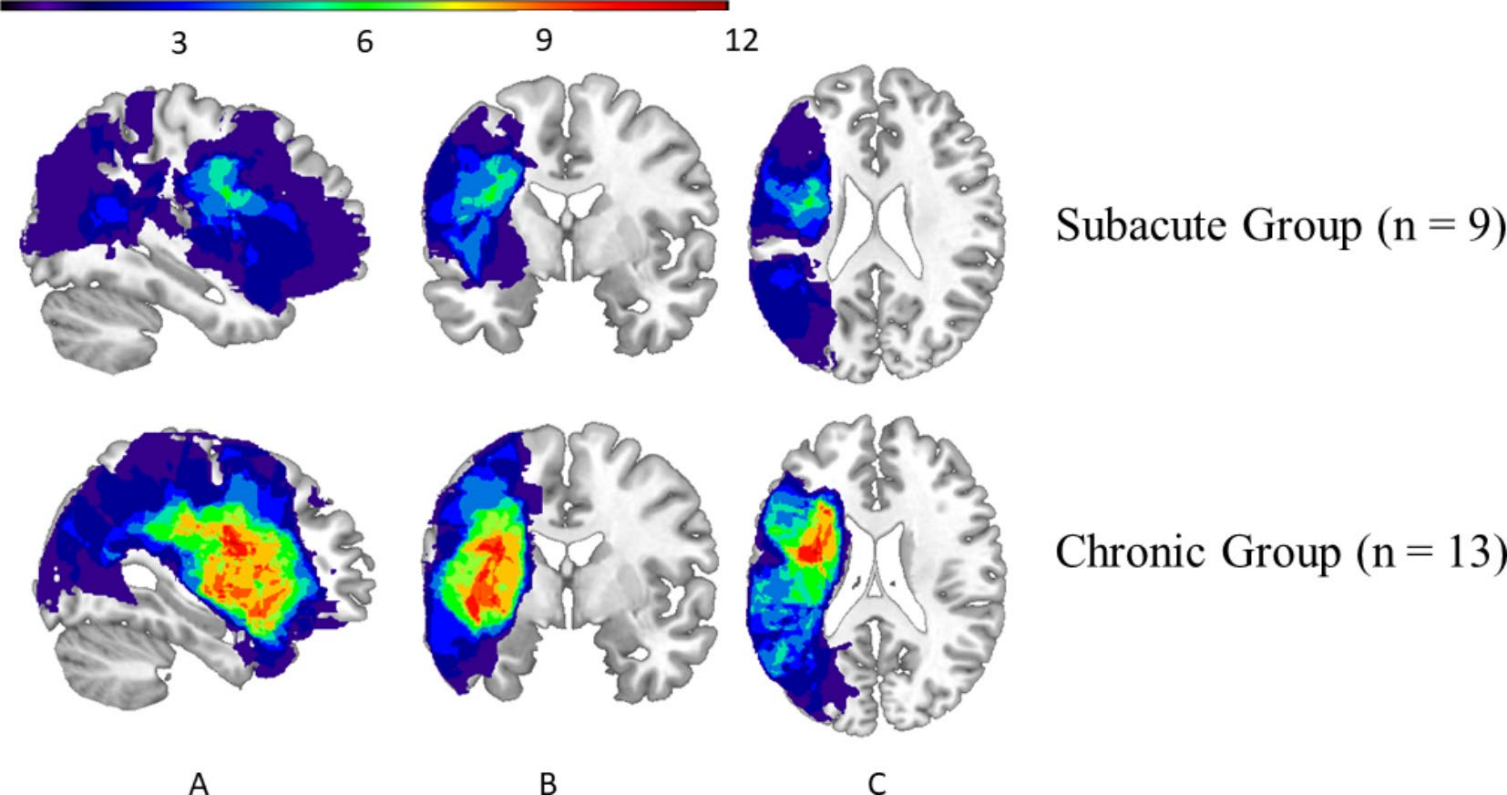
Overlay of the participants’ normalized lesion reconstructions on a common brain template in three sections: (**A**) sagittal, (**B**) coronal, and (**C**) axial in Subacute and Chronic Groups. Brighter areas (red, orange, yellow, and green) indicate regions of greater overlap, while darker areas (blue and dark blue) indicate lesser overlap.

The location of the lesion was verified by CT or MRI in 22 out of 27 individuals—9 in the Subacute Group and 13 in the Chronic Group (Fig. 1). Neuroanatomical analyses using MRIcroN and SPM12 confirmed that lesions in both groups were localized only in the left hemisphere. The total lesion volume in the Chronic Group was significantly larger than in the Subacute Group (see Table 2). In the Subacute Group, the lesion most commonly affected: the insular cortex, postcentral gyrus, central operculum cortex, middle frontal gyrus, inferior frontal gyrus, precentral gyrus, planum polare, planum temporale, and Heschl’s gyrus. In the Chronic Group, the most commonly affected structures were: the insular cortex, precentral gyrus, central operculum cortex, inferior frontal gyrus, temporal pole, postcentral gyrus, frontal operculum cortex, planum temporale, and the putamen. The most frequent lesion areas in both groups are presented in Table 3. Fourteen of these structures were similarly affected in both groups, whereas five of them (orbitofrontal cortex, temporal pole, putamen, amygdala, and pallidum; marked in gray in Table 3) were at least twice as frequently affected in chronic patients as in subacute patients.

**Table 3:** Percent of patients with affected brain structures.

Brain structure	% of patients	
	Subacute group (*n* = 9)	Chronic group (*n* = 13)
Insular Cortex	89	92
Postcentral Gyrus	89	85
Central Operculum Cortex	89	92
Inferior Frontal Gyrus, Pars Opercularis	67	85
Middle Frontal Gyrus	67	69
Planum Temporale	67	85
Planum Polare	67	77
Heschl’s Gyrus	67	77
Precentral Gyrus	67	92
Parietal Operculum Cortex	63	69
Anterior Supramarginal Gyrus	56	69
Frontal Operculum Cortex	56	85
Inferior Frontal Gyrus, Pars Triangularis	44	62
Supramarginal Gyrus, Posterior Division	44	31
Frontal Orbital Cortex	33	69
Temporal Pole	33	85
Putamen	22	85
Amygdala	22	62
Pallidum	11	62

### Ethical approval

This study was approved by the Ethical Commission at the University of Social Sciences and Humanities (permission no 26/2017, registered as 35/2017) and was in accordance with the ethical standards of the Helsinki Declaration. All patients or their caregivers provided written informed consent to participate in the study prior to testing.

### Procedure

#### Go/No-Go task

The visual Go/No-Go task was designed using Presentation software, version 14.9 (Neurobehavioural Systems Inc.). The experimental stimuli were two black shapes—a triangle and a circle. The shapes were presented on a gray background in the center of the 22” screen. The monitor was located at a distance of approx. 85 cm from the participant.

The participant’s task was to press a button on a response pad (Cedrus RB-834, Cedrus Corporation, San Pedro, USA) when the triangle (Go stimulus) was presented and to inhibit pressing when the circle (No-Go stimulus) was presented. 

The procedure was divided into 5 blocks of 60 trials each (75% Go and 25% No-Go stimuli). The stimuli were presented in a pseudo-randomized order and displayed for 300 ms. Three inter-stimulus-intervals (800 ms, 1000 ms, and 1200 ms) were applied. Before the task proper, the participants completed an introductory session to familiarize themselves with the task.

Outcome measures: reaction time (RT) on Go stimuli as well as d prime (the sensitivity index) was used to evaluate participants’ performance^29^. The d prime index is the difference between Z transforms of hit rate and false alarms. Hit rate is defined as the proportion of hits when a signal is present to all target stimuli and false alarms represent the proportion of responses when a signal is absent to all non-target stimuli. Perfect scores were adjusted according to following formulas: 1 – 1/(2n) for perfect hits, and 1/(2n) for zero false alarms, where n was the number of total hits or false alarms.

#### EEG analyses

##### Recording and data preprocessing

EEG was recorded with 32 electrodes referenced to the electrode placed on the nose in an extended 10–20 system re-referenced to the FCz for connectivity analyses. Preprocessing included epoching the signal into 800 ms windows spanning a period 300 ms before stimulus onset and 500 ms after onset, 1–45 Hz filtering (Hamming windowed FIR filter with heuristic order/transition band width estimation implemented in EEGlab), and exclusion of data epochs containing large muscle and movement artifacts detected by visual inspection. Channels with excessive artifacts or missing signal were interpolated with a spherical interpolation method^30^ using the EEGlab function (pop_interp). 

##### Connectivity analyses

In order to evaluate the impact of the recovery phase on neural connectivity, we employed corrected imaginary Phase Locking Value (ciPLV). CiPLV, is modification of phase locking value originally proposed by Lachaux^31^. The assumption behind the PLV and ciPLV is signal synchronization: it looks for latencies at which the phase difference between the signals varies little across trials. The original PLV metrics has been frequently used for assessing connectivity in EEG/MEG studies, as exemplified by research conducted by Vanden Bosch der Nederlanden et al.^32^, Gong et al.^33^, Neubauer and Fink^34^ and Varela et al^35^. An advantage of the PLV and its ciPLV counterpart over the other methods is its robustness in the presence of noisy signals^36^. Modification made by Bruña and colleagues^37^ introduced normalization to imaginary PLV proposed earlier by Nolte et al.^38^ making it robust to the presence of volume conduction or source leakage effects while preserving other PLV properties. A pairwise comparison between Chronic and Subacute Groups was performed on segmented (epoched) signals of all electrode pairs across the following frequency bands: theta (4–7 Hz), alpha (8–12 Hz), beta (15–30 Hz), and gamma (31–45 Hz). Only data from valid No-Go trials were included in this analysis. Cluster-level statistical permutation tests implemented in the MNE-python toolkit^39^ were used to compare connectivity matrices between groups.

Finally, to evaluate the effect of stroke phase on the overall organization of the brain EEG networks, we applied global graph theory metrics implemented in python toolbox bctpy (https://pypi.org/project/bctpy/) and in-house python scripts. Data preprocessing was performed with the EEGLAB toolbox^40^. In order to identify brain network organization differences during recovery phases we compared efficiency, modularity and clustering coefficients. All three metrics are commonly used to assess optimal structure of the network and to analyze changes in brain network organization (both EEG and fMRI) after stroke or during rehabilitations^41,42^. Here we used local (at the electrode level) metrics to detect differences in brain regions and their global counterparts to find out how local differences affect the overall functioning of the brain network.

Group comparisons for the local graph theoretic metrics were performed using a cluster-level statistical permutation implemented in the MNE-python toolkit^39^. Group comparisons for the global graph theoretic metrics and behavioral measures were performed using a two-tailed Mann–Whitney* U*-test and the relationships between variables were explored by Spearman correlation analyses. To mitigate the effect of outliers on correlation analyses, prior to calculation of Spearman correlations, d prime outliers were removed. A value was considered an outlier if it was more than three scaled median absolute deviations from the median. Results for all statistical analyses were considered significant at the threshold of* p* < 0.05.

## Results

### Behavioural results of the Go/No-Go task

#### Lesion volume and performance in the Go/No-Go Task

The volume of the lesion showed no correlation with reaction time nor with d prime on the Go/No-Go task for neither the Chronic or Subacute Groups.

#### Group differences in the Go/No-Go Task performance

**Fig. 2:**
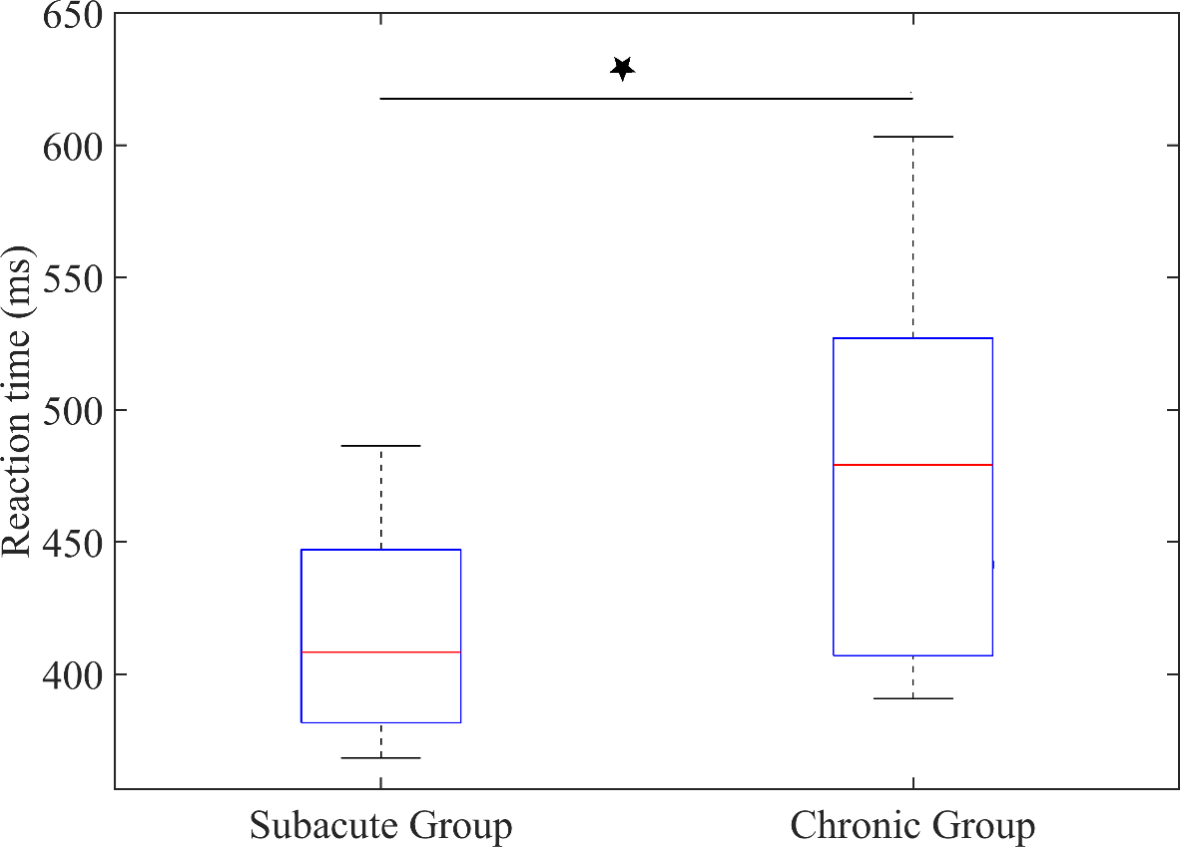
Differences in reaction time performance between Subacute and Chronic Groups. Asterisk denotes significance at* p* = 0.020 for the Mann–Whitney* U*-test.

Comparison of behavioral performance showed significantly shorter reaction time for the Subacute Group than the Chronic Group (Mann–Whitney test,* U* = 43,* p* = 0.020, Fig. 2) and no significant differences for d prime (Mann–Whitney test,* U* = 57.5,* p* = 0.104)

### Connectivity and network analyses

#### Group differences in brain functional connectivity

**Fig. 3:**
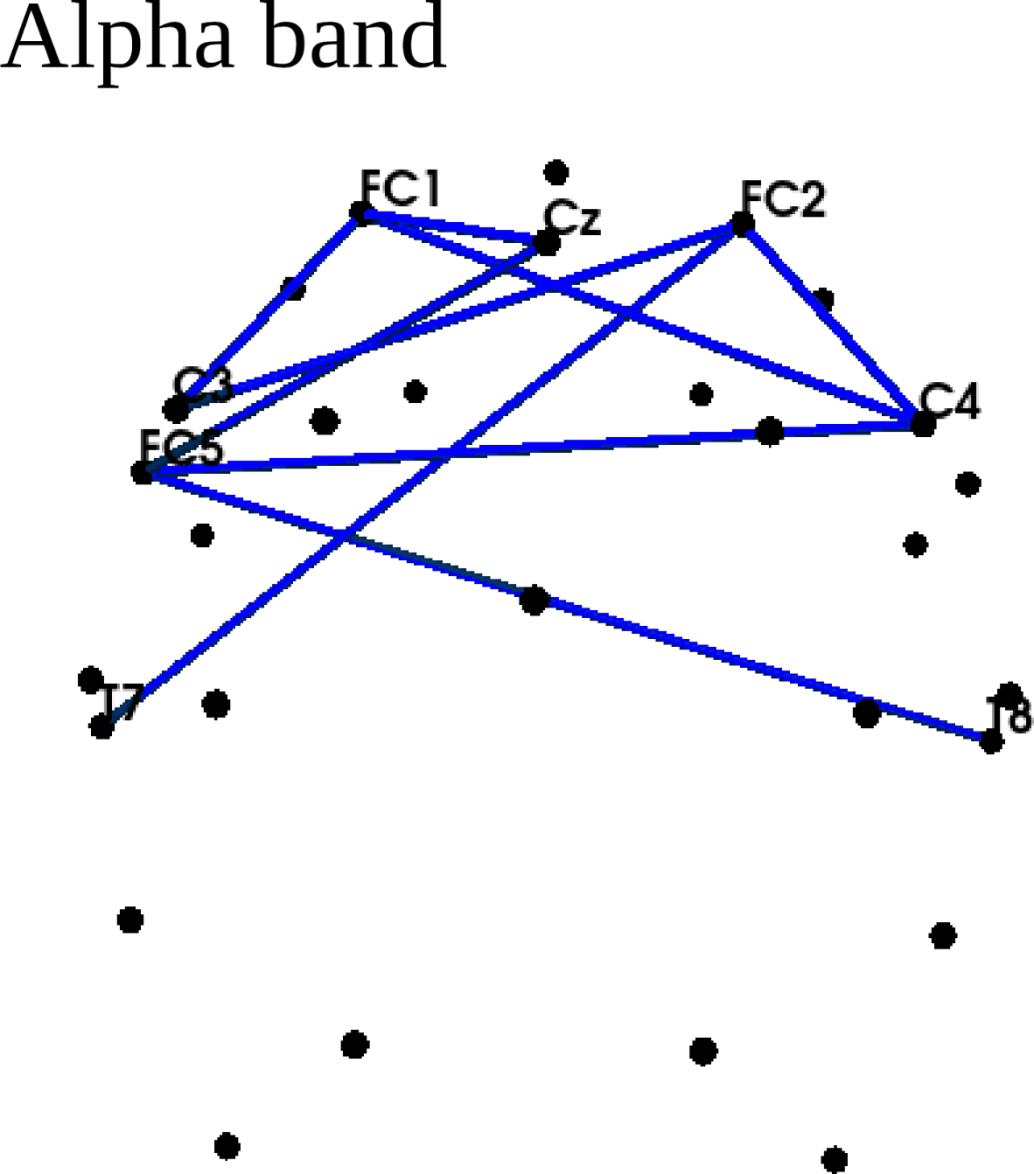
Connectivity differences between Chronic and Subacute Groups, approximated by corrected imaginary phase locking value (ciPLV). A significant cluster (*p* < 0.05) was found only in the alpha band. Blue lines indicate lower connectivity strength in the Subacute Group.

Connectivity analyses revealed a significant cluster only in the alpha band (Fig. 3) showing lower connectivity strength in the Subacute Group. The differences were found in the connections spanning left and right frontal and temporal regions.

Recovery processes taking place both in Subacute and Chronic Groups could evoke brain organization changes undetected by connectivity analyzes, thus in the next step we performed brain network organization analyzes using graph theoretic methods at local (individually for each electrode) and global (for all electrodes together) level. 

#### Group differences in brain network organization

**Fig. 4:**
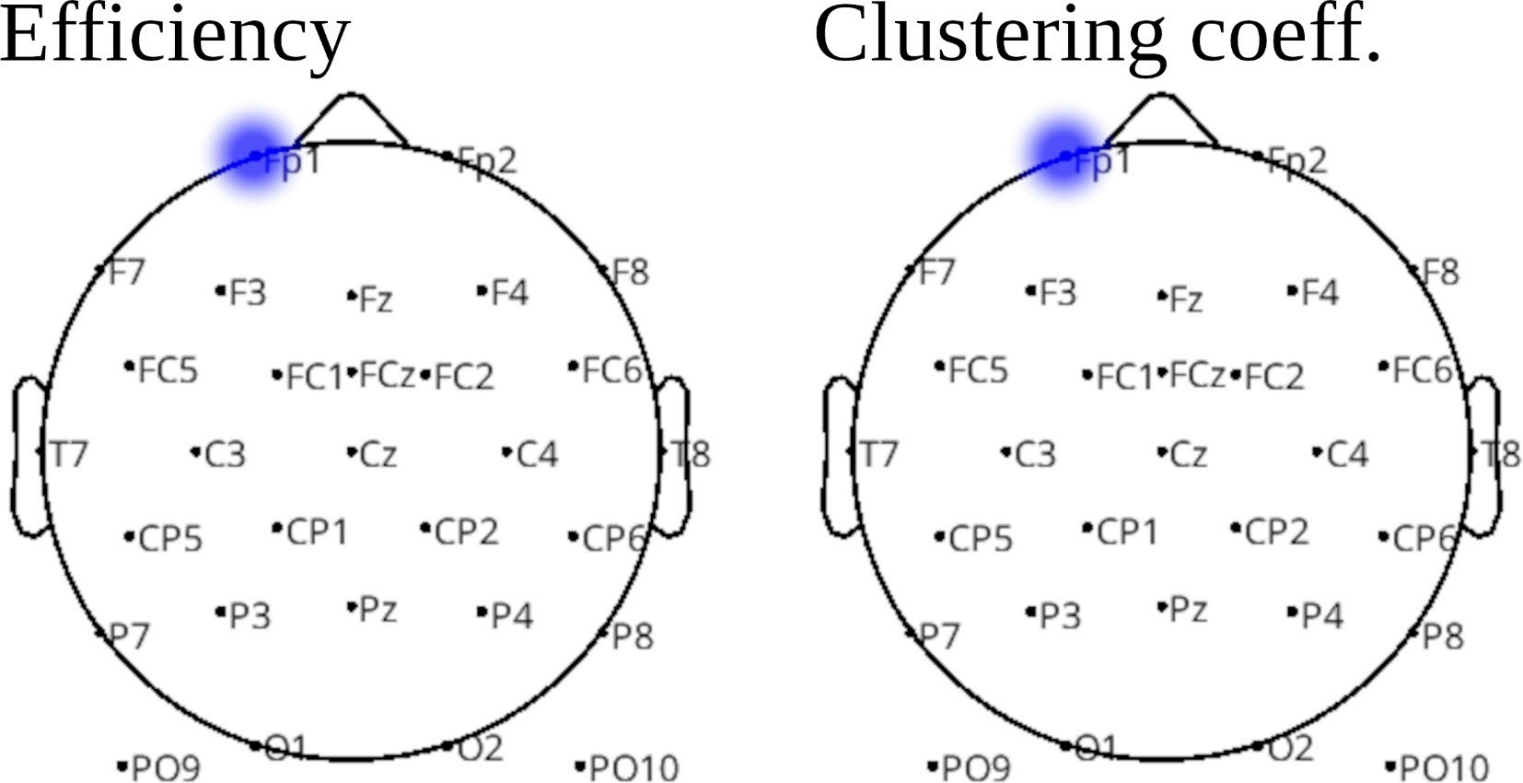
Group comparisons for the local efficiency and clustering coefficient. Blue color indicates significantly (*p* < 0.05) lower value of both metrics in the Subacute Group. Comparisons were performed using cluster-level statistical permutation test.

Analyses using local graph theoretic metrics indicated lower efficiency and clustering coefficient in the Subacute Group at the Fp1 electrode in alpha band (Fig. 4). Taking as the baseline values in the Subacute Group, the efficiency and clustering coefficient calculated with the ciPLV were lower in the Subacute Group by −0.26 and −0.28 respectively. 

**Fig. 5:**
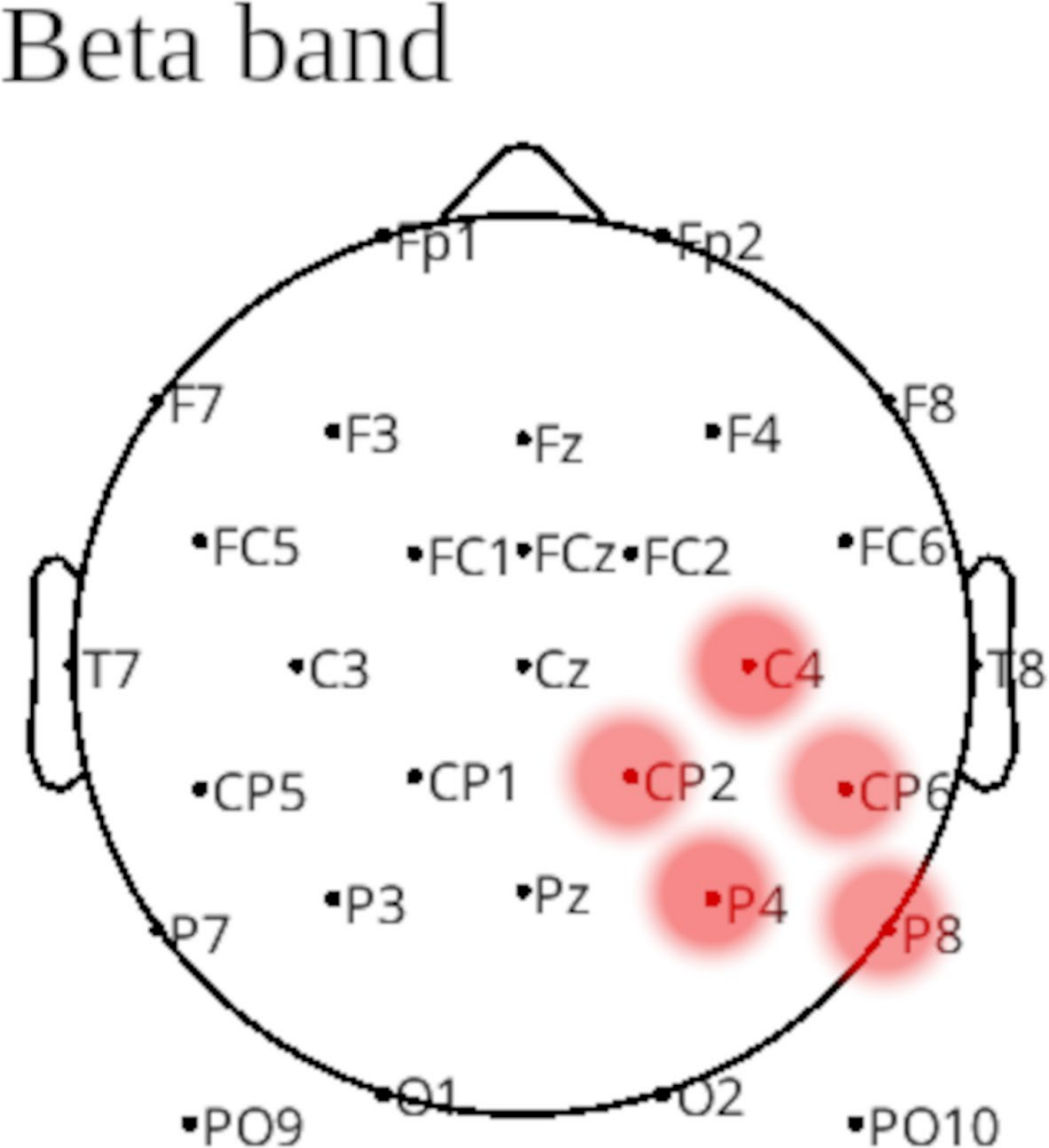
Group differences in the modularity in beta band. Red color indicates significantly (*p* < 0.05) higher modularity in the Subacute Group. Comparisons were performed using cluster-level permutation tests.

Furthermore, group differences in the beta band showed significantly higher local modularity values in the Subacute Group at electrodes located in the right centro-parietal and parietal regions (Fig. 5).

Although local indices of graph theoretic metrics showed lower efficiency in the left frontal region in the alpha band (Fig. 5), the analysis of global measures accounting for global brain network organization showed a different picture, i.e., higher global efficiency and lower clustering coefficient were identified in the Subacute Group compared to the Chronic one (Table 4).

**Table 4:** Differences between the Subacute and Chronic Groups in the global graph theoretic metrics. Numbers denote the difference in percentage in respect to the Subacute Group. Positive values indicate higher metrics in the Subacute Group, negative - in the Chronic one. All shown differences are significant at *p* < 0.05 Mann–Whitney* U* test.

	% of diff in relation to the Subacute Group		
Metrics	Efficiency	Modularity	Clustering
Alpha	18.1	–	−33.4

## Discussion

In the current study, we aimed to explore EEG synchronization and network organization patterns associated with behavioral performance in an attentional Go/No-Go task in people with aphasia in both subacute and chronic stroke phases. The obtained results allowed us to identify certain behavioral and EEG network differences between groups in different stroke phases.

The behavioral domain results revealed:

(I) Significantly shorter reaction time in performance of the Go/No-Go task in the Subacute Group compared to the Chronic Group (Fig. 2).
(II) No correlation between lesion volume and reaction time and between lesion volume and d prime in the Go/No-Go task for either the Chronic or Subacute Groups.

One of the most interesting behavioral findings of our study was the lack of correlation between lesion volume and performance in both groups and, contrary to our expectations, shorter reaction times were present in the Subacute than in the Chronic Group. Both of those observations could be explained by the smaller percentage of patients with putamen lesions in the Subacute than in the Chronic Group (Table 3). The putamen is one of the structures involved in stimulus response^43,44^. It may be involved in resolving competition between the explicit rule-based system and acting based on implicit procedural learning^45,46^, and lesions in this area can lead to deficits in executive functioning, verbal short-term memory, and working memory^47^. Putamen–caudate lesions may also impair delayed response, altering behavior and visual discrimination learning^43,48,49^. Thus, the higher percentage of patients with putamen lesions in the Chronic Group could have resulted in their longer average reaction time.

The neuronal underpinning of the observed behavioral performance included:

(I) Weaker connectivity in the alpha band in the Subacute Group spanned left and right frontal and temporal regions (Fig. 3).
(II) Lower local efficiency and clustering coefficients in the alpha band in the Subacute Group (Fig. 4).
(III) Higher local modularity in the beta band in the Subacute Group (Fig. 5).
(IV) Higher global efficiency and lower clustering coefficient in the Subacute Group (Table 4).

Weakened synchronization in the alpha band may indicate desynchronization of the attentional process between the hemispheres. Low alpha band power is associated with activations observed in fMRI in frontal and parietal structures, which are involved in attention^50^. Lower interhemispheric alpha synchronization in frontal areas may therefore indicate that attentional processes in the two hemispheres run at least partially independently of each other. This desynchronization may be the result of global (whole brain) compensatory processes. The relevance of this hypothesis may be confirmed by the fact that the Subacute Group, of lower local efficiency in the alpha band (Fig. 4) showed higher global efficiency (Table 4), characterized by more effective information processing of the neuronal network^51^.

Referring to compensatory processes, higher modularity of the unaffected right parietal regions in the beta band in the Subacute Group (Fig. 5) may indicate another aspect of these mechanisms. Task performance requires synchronized activity of many involved structures^28^, which during resting state conform to highly modular (segregated) organization. The synchrony in the beta band is theorized to be crucial for both cognitive and motor functions. While the beta band activity is primarily linked with attention^52^, its coherence might indicate the interaction between different brain regions necessary to successful performance in Go/No-Go task^53,54^. Therefore, segregation of the brain networks involved in the task need to be broken down in order to align activity of many involved structures^28^. This hypothesis was confirmed by Bola & Sabel^55^ who found decreased network modularity in theta and beta bands during successful task performance. Consequently, increased modularity in the beta band in the Subacute Group on the side opposite to lesion might suggest a broad inhibition of attentional processes. Similar to the inhibition of motor responses, an increased modularity in the beta band in the healthy hemisphere could serve to equalize the cognitive abilities of the two hemispheres, preventing exacerbation of the patient’s conditions caused by large differences in neuronal synchronization between healthy and affected hemispheres. The negative impact of differences in synchronization seems to be confirmed by the study of Kelly et al.^56^, who postulate that one of the likely causes of dyslexia may be the lack of synchronization between the brain hemispheres.

Interestingly, local deficiencies in the brain network organization of the Subacute Group in respect to the Chronic one such as lower local efficiency in left frontal areas (Fig. 4) and higher modularity of the right centro-parietal and parietal areas (Fig. 5) seem to contribute to overall more efficient global network organization manifested by its higher global efficiency and lower clustering coefficient (Table 4). This observation was further confirmed by shorter reaction times in the Subacute Group than in the Chronic one.

In conclusion, the identified differences in the organization of neuronal networks between the groups, covering both the affected and unaffected hemispheres, may serve to re-synchronize neuronal activity and cognitive processes disrupted by the stroke.

### Limitations

The biggest limitation of the current study is that it compares functional connectivity during different stroke phases in two separate groups of participants. Despite strict inclusion criteria (see Participants), our groups differed in some variables, such as lesion volume. Conducting a longitudinal study with a single group of participants being tested first in the subacute phase and then again in the chronic phase would exclude between-group differences and thereby improve the reliability of the results. However, based on our previous long-term experience, huge drop-out is always observed in longitudinal studies as patients may suffer from additional medical incidents (i.e., recurrent stroke, epileptic seizures) that may affect their cognitive status and prevent them from continuing in the study.

## Conclusions

We postulate that desynchronization of the frontal areas of the hemispheres and local changes in the organization of brain networks, such as increased modularity in the unaffected hemisphere or lower network efficiency in the left frontal area, may be a natural mechanism to ensure reorganization and resynchronization of the cerebral hemispheres after stroke to enable recovery of inhibitory processes. If confirmed, these observations should be taken into account in the development of new rehabilitation therapies.

## Data Availability

The datasets generated and/or analysed during the current study are available from the corresponding author on request.
